# Increased atherosclerosis in a mouse model of glycogen storage disease type 1a

**DOI:** 10.1016/j.ymgmr.2022.100872

**Published:** 2022-04-21

**Authors:** Anouk M. La Rose, Anouk G. Groenen, Benedek Halmos, Venetia Bazioti, Martijn G.S. Rutten, Kishore A. Krishnamurthy, Mirjam H. Koster, Niels J. Kloosterhuis, Marieke Smit, Rick Havinga, Gilles Mithieux, Fabienne Rajas, Folkert Kuipers, Maaike H. Oosterveer, Marit Westerterp

**Affiliations:** a Department of Pediatrics, University Medical Center Groningen, University of Groningen, Groningen, the Netherlands; b Université Claude Bernard Lyon 1, Université de Lyon, INSERM UMR-S1213, Lyon, France; c Department of Laboratory Medicine, University Medical Center Groningen, University of Groningen, Groningen, the Netherlands

**Keywords:** Glycogen storage disease type 1a, Atherosclerosis, Hyperlipidemia, GSD Ia, Glycogen storage disease type 1a, G6PC1, Glucose-6-phosphatase enzyme, G6P, Glucose-6-phosphate, HDL, High-density lipoprotein, *Ldlr*^−/−^, LDL receptor deficient, SFA, Saturated fatty acids, PUFA, Poly-unsaturated FA, TG, Triglycerides, VLDL, Very-low-density lipoprotein, WTD, Western-type diet

## Abstract

Glycogen storage disease type 1a (GSD Ia) is an inborn error of carbohydrate metabolism. Despite severe hyperlipidemia, GSD Ia patients show limited atherogenesis compared to age-and-gender matched controls. Employing a GSD Ia mouse model that resembles the severe hyperlipidemia in patients, we here found increased atherogenesis in GSD Ia. These data provide a rationale for investigating atherogenesis in GSD Ia in a larger patient cohort.

## 1 Introduction

Glycogen storage disease type 1a (GSD Ia) is an inborn error of carbohydrate metabolism, caused by mutations in the gene encoding the catalytic subunit of glucose-6-phosphatase enzyme (*G6PC1*) [1]. *G6PC1* is expressed by liver, kidney, and intestine, and is essential for conversion of glucose-6-phosphate (G6P) into glucose [2]. GSD Ia patients show severe hypoglycemia upon fasting and show 10-fold higher plasma very-low-density lipoprotein (VLDL)-triglycerides (TG) and 2-fold higher VLDL- and LDL-cholesterol compared to age-and-gender matched controls [3,4].

Despite severe hyperlipidemia, GSD Ia patients show a ~ 10% reduction in carotid intima media thickness compared to age-and-gender matched controls [5], indicative of decreased atherosclerotic lesion size in the carotid arteries. This decrease has been attributed to LDL particles being less susceptible to oxidation [4], due to increased saturated fatty acids (SFA) in plasma VLDL/LDL [4]. However, diets rich in SFA are associated with increased atherosclerosis in humans [6], and replacement of SFA with polyunsaturated fatty acids decreases atherosclerosis in mice [7]. Therefore, the decrease in atherosclerosis in GSD Ia cannot be explained by increased plasma SFA levels. Further research into the mechanisms driving the decreased atherogenesis in GSD Ia patients is thus needed. We previously employed hepatocyte-specific *G6pc* deficient mice as a model of GSD Ia. Although these mice are slightly hyperlipidemic during fasting, their plasma cholesterol mainly circulates in high-density lipoproteins (HDL) and not in VLDL or LDL [8]. This is similar to wild-type mice. For this reason wild-type mice do not develop atherosclerosis [9]. For atherosclerosis studies, mice deficient in the LDL receptor (*Ldlr*^*−/−*^ mice) have been generated [10]. When fed a cholesterol-rich Western-type diet (WTD), *Ldlr*^*−/−*^ mice show high levels of VLDL/LDL-cholesterol, and develop advanced atherogenesis with plaques comparable to humans [9]. Therefore, to study atherogenesis under hyperlipidemic conditions similar to those in GSD Ia patients, we generated hepatocyte-specific *G6pc* deficient mice on the *Ldlr*^−/−^ background and fed these mice WTD.

## 2 Methods

### 2.1 Animals

*B6.G6pc*^*lox/lox*^ and *B6.G6pc*^*lox/lox*^*.SA*^*CreERT2*^ mice were intercrossed with *Ldlr*^*−/−*^ (stock 002207; Jackson Laboratories, Bar Harbor, ME, USA) mice to generate *B6.G6pc*^*lox/lox*^*Ldlr*^*−/−*^ and *B6.G6pc*^*lox/lox*^*.SA*^*CreERT2*^ *Ldlr*^*−/−*^ mice. At 8–12 weeks of age, mice received intraperitoneal tamoxifen injections (T5648; Sigma-Aldrich, St. Louis, MO, USA) (1 mg/day in 95% sunflower oil/5% ethanol) for five consecutive days to induce hepatocyte-specific *G6pc* deficiency, as described previously [11]. We refer to *B6.G6pc*^*lox/lox*^*.SA*^*CreERT2*^*Ldlr*^*−/−*^ and littermate *B6.G6pc*^*lox/lox*^*Ldlr*^*−/−*^ mice as L*-G6pc*^*−/−*^*Ldlr*^*−/−*^ and *Ldlr*^*−/−*^ mice. Mice were housed in a light (lights on at 7:00 AM, lights off at 7:00 PM) and temperature (21 °C)-controlled facility and had ad libitum access to water and food. After tamoxifen injections, male and female mice were fed a chow diet (RMH-B, AB diets, Woerden, The Netherlands) for 4 weeks (recovery period), followed by WTD (40% fat, 0.15% cholesterol; D12079B, Research Diets, New Brunswick, NJ, USA) for 8 or 15 weeks. Mice were randomly assigned to experimental groups. The number of mice used for each experiment and the period of WTD feeding are indicated in the figure legends. No inclusion or exclusion criteria were used. Experiments were performed at 8:00 AM in fed condition or at 2:00 PM after a 6 h fasting period during the inactive period. Mice were sacrificed after 8 (~20 weeks old) or 15 (~27 weeks old) weeks of WTD feeding. All animal studies were approved by the Institutional Animal Care and Use Committee from the University of Groningen under permit number AVD105002015244 and adhered to guidelines set out in the 2010/63/EU directive.

### 2.2 Plasma lipoprotein analysis

Blood samples were collected by tail bleeding into EDTA-coated tubes. Plasma was separated by centrifugation and plasma cholesterol and TG levels were measured using enzymatic kits (113,009,910,026 and 157,109,910,917, respectively; Diasys Diagnostic Systems, Holzheim, Germany) with Cholesterol FS or Precimat Glycerol standard (113,009,910,030; Diasys Diagnostic Systems and 10,166,588; Roche, Mannheim, Germany, respectively) for the calibration curve. Lipoprotein cholesterol and triglyceride distribution were measured by fast performance liquid chromatography (FPLC) using a system containing a PU-4180 pump with a linear degasser and UV-4075 UV/VIS detectors (Jasco, Tokyo, Japan). Pooled plasma samples were injected onto a Superose 6 Increase 10/300 GL column (GE Healthcare, Hoevelaken, The Netherlands) and eluted at a constant flow rate of 0.31 mL/min in PBS (pH 7.4). Cholesterol or triglycerides were measured in line by addition of cholesterol or triglyceride reagent at a constant flow rate of 0.1 mL/min using an additional PU-4080i infusion pump (Jasco, Tokyo, Japan). Data acquisition and analysis were performed using ChromNav software (version 1.0; Jasco, Tokyo, Japan).

### 2.3 Atherosclerotic lesion analysis

Female *Ldlr*^−/−^ and L*-G6pc*^−/−^*Ldlr*^*−/−*^ mice were fed a WTD for 8 weeks and males for 15 weeks. Mice were sacrificed, hearts were isolated and fixed in 4% phosphate buffered paraformaldehyde, embedded in paraffin, and 4 μm sections of the aortic root area were made and stained with hematoxylin-eosin (H&E). Atherosclerotic lesion area was quantified using Image J software (NIH) and the average of 5 sections with 40 μm distance between the sections was calculated for each mouse.

### 2.4 Plasma uric acid measurement

Blood samples were collected by tail bleeding into EDTA-coated tubes. Plasma was separated by centrifugation and uric acid levels were measured using a uric acid kit (KA1651, Abnova, Tapei, Taiwan) according to the manufacturer's instructions.

### 2.5 Statistical analysis

All data are presented as mean ± SEM. The unpaired *t*-test was used to compare two datasets. Group size and statistical test are reported in the figure legends. The criterion for significance was set at *P* < 0.05. Statistical analysis was performed using GraphPad Prism 5.

## 3 Results

**Fig. 1:**
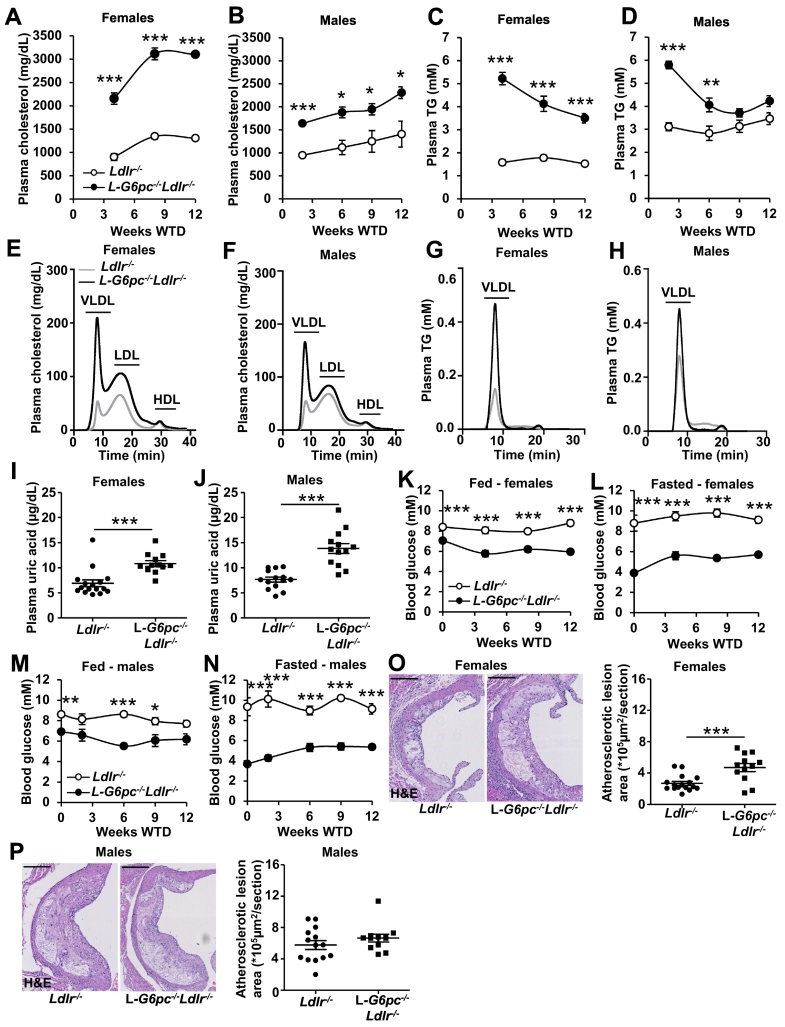
**Hepatocyte-specific *G6pc1* deficiency increases plasma lipids and uric acid levels in female and male *Ldlr***^***−/−***^**mice fed Western-type diet, and increases atherosclerotic lesion area in females.** Hepatocyte-specific *G6pc1* deficiency was induced by tamoxifen injections. After a recovery period of 4 weeks on chow diet, female and male L-*G6pc*^*−/−*^*Ldlr*^−/*−*^ and *Ldlr*^−/*−*^ mice were fed Western-type diet (WTD). Blood was collected after a 6 h fast (8:00 AM -2:00 PM) at the indicated time points on WTD and plasma cholesterol and triglyceride (TG) levels were determined. Lipoprotein fractions from pooled plasma samples (*n* = 8–15 per pool) were separated using fast performance liquid chromatography. (A, B) Plasma cholesterol levels in female (A) and male (B) L-*G6pc*^*−/−*^*Ldlr*^−/*−*^ and *Ldlr*^−/*−*^ mice. (C, D) Plasma TG levels in female (C) and male (D) L-*G6pc*^*−/−*^*Ldlr*^−/*−*^ and *Ldlr*^−/*−*^ mice. Data are shown as mean ± SEM. (E, F) Lipoprotein cholesterol distribution (8 weeks WTD) in female (E) and male (F) L-*G6pc*^*−/−*^*Ldlr*^−/*−*^ and *Ldlr*^−/*−*^ mice. (G, H) Lipoprotein TG distribution (8 weeks WTD) in female (G) and male (H) L-*G6pc*^*−/−*^*Ldlr*^−/*−*^ and *Ldlr*^−/*−*^ mice. (I, J) Plasma uric acid levels in female L-*G6pc*^*−/−*^*Ldlr*^−/*−*^ and *Ldlr*^−/*−*^ mice at 8 weeks WTD (I) and male L-*G6pc*^*−/−*^*Ldlr*^−/*−*^ and *Ldlr*^−/*−*^ mice at 15 weeks WTD (J). (K–N) At the indicated time points on WTD blood glucose levels were measured in the fed (8:00 AM) (K, M) and fasted (6 h fast from 8:00 AM - 2:00 PM) (L,N) condition in female (K,L) and male (M, N) L-*G6pc*^*−/−*^*Ldlr*^−/*−*^ and *Ldlr*^−/*−*^ mice. (*n* = 5–6 for males, *n* = 11–12 for females). (O, P) L-*G6pc*^*−/−*^*Ldlr*^−/*−*^ and *Ldlr*^−/*−*^ female mice were sacrificed after 8 weeks of WTD (O) and L-*G6pc*^*−/−*^*Ldlr*^−/*−*^ and *Ldlr*^−/*−*^ male mice after 15 weeks WTD (P). Hearts were isolated, sections were made of the aortic root, stained with hematoxylin-eosin (H&E) and atherosclerotic lesion area was quantified. Representative examples are shown. Scale bar represents 200 μm. (I, J, O, P) Each data point represents an individual mouse. (*n* = 13–14 for males, *n* = 12–16 for females). (I–P) Data are shown as mean ± SEM. *p < 0.05, **p < 0.01, ***p < 0.001, by *t*-test.

To investigate the role of hepatocyte-specific *G6pc* in atherogenesis, we generated L-*G6pc*^*−/−*^*Ldlr*^*−/−*^ mice and *Ldlr*^*−/−*^ littermate controls. On the *Ldlr*^*−/−*^ background, hepatocyte-specific *G6pc* deficiency increased plasma cholesterol levels during WTD feeding by ~2.3-fold in female and by ~1.7-fold in male *Ldlr*^*−/−*^ mice (Fig. 1A, B). Plasma cholesterol levels increased further over the course of WTD feeding, while the difference between the genotypes remained similar (Fig. 1A, B). Hepatocyte-specific *G6pc* deficiency increased plasma TG by ~2.3-fold in females and by ~1.9-fold in males at 2 weeks of WTD (Fig. 1C, D). These increases gradually declined during the study and were no longer different for male mice at 9 weeks of WTD (Fig. 1C, D). Sharing similarities with findings in GSD Ia patients, the increases in plasma lipids were reflected by an increase in VLDL- and LDL-cholesterol, as well as VLDL-TG (Fig. 1E-H). The decreased susceptibility of LDL to oxidation in GSD Ia patients has been attributed to elevated plasma concentrations of uric acid [12]. Consistently, L-*G6pc*^*−/−*^*Ldlr*^*−/−*^ mice showed increased plasma uric acid levels compared to *Ldlr*^*−/−*^ female and male mice fed WTD (Fig. 1I, J). We then measured blood glucose levels in mice fed chow or WTD. Similar to previous data [11,13], hepatocyte-specific *G6pc1* deficiency induced hypoglycemia (blood glucose ≤4.0 mM) upon a 6 h fasting period (Fig. 1K-N). However, after 2 weeks of WTD, L-*G6pc*^−/−^*Ldlr*^*−/−*^ mice showed blood glucose levels of ~5.5 mM in both fed and fasted conditions (Fig. 1K-N). This is likely due to the WTD being rich in sucrose (35% sucrose). Sucrose rapidly increases blood glucose levels to promote insulin release [14]. When fed chronically, sucrose lowers blood glucose levels in L-*G6pc*^*−/−*^ mice as also shown upon high fat/high sucrose (HF/HS) diet feeding in a previous study [15].

We subsequently assessed atherosclerotic lesion area in these mice. In line with guidelines for atherosclerosis studies [9], we evaluated lesion area in mice of both genders. Given that atherosclerosis develops faster in female *Ldlr*^*−/−*^ mice than in males [9], we fed female L-*G6pc*^*−/−*^*Ldlr*^*−/−*^ and *Ldlr*^*−/−*^ mice WTD for 8 weeks, and used 15 weeks of WTD for males. In contrast to findings on carotid intima media thickness in humans [5], but in line with the increases in plasma lipid levels, hepatocyte-specific *G6pc* deficiency increased atherosclerotic lesion area by ~1.5-fold in female *Ldlr*^*−/−*^ mice after 8 weeks of WTD (Fig. 1O). Male *Ldlr*^*−/−*^ mice fed WTD for 15 weeks showed larger atherosclerotic plaques than expected based on earlier studies [16]. Hepatocyte-specific *G6pc* deficiency tended to increase atherosclerotic lesion area in *Ldlr*^*−/−*^ males fed WTD (Fig. 1P). As a consequence of the longer WTD feeding in males and the lesions in *Ldlr*^*−/−*^ mice being larger than usual, we may have missed detecting a significant increase of hepatocyte-specific *G6pc* deficiency on atherosclerotic plaques, though we observed a tendency that did not reach statistical significance (*p* = 0.10). Together, hepatocyte-specific *G6pc* deficiency increases plasma lipid levels, which increases atherogenesis, at least in female WTD-fed *Ldlr*^*−/−*^ mice, and shows a tendency to an increase in lesion size in males.

## 4 Discussion

GSD Ia patients show a ~ 10% decrease in carotid intima media thickness compared to age-and-gender matched controls [5], despite hyperlipidemia reflected by 10-fold higher plasma TG and 2-fold higher cholesterol levels [4,17]. Even though the patient cohort was small (*n* = 9 per group), these findings suggest that GSD Ia patients are less susceptible to atherogenesis [5]. To investigate mechanisms underlying this observation, we bred L-*G6pc*^*−/−*^ mice on the *Ldlr*^−/*−*^ background and fed them a cholesterol-rich WTD to induce hyperlipidemia and atherosclerosis. Similar to GSD Ia patients, hepatocyte-specific *G6pc* deficiency increased plasma lipids in mice of both genders, and increased plasma uric acid levels. However, unlike in GSD Ia patients, hepatocyte-specific *G6pc* deficiency increased atherosclerotic lesion size in female WTD-fed *Ldlr*^*−/−*^ mice, while showing a tendency towards an increase in males.

Our data suggest, in contrast to findings in GSD Ia patients [5], that hyperlipidemia in a mouse model of GSD Ia does accelerate atherosclerosis. We also found that hepatocyte-specific *G6pc* deficiency increased plasma uric levels. While this increase is in line with findings in GSD Ia patients, it has been suggested that elevated plasma uric acids in GSD Ia decreases LDL oxidation, and may thus be athero-protective [12]. In contrast, several epidemiological studies and studies in animal models have shown that uric acid levels are associated with an increase in atherosclerosis [18]. These studies [18] thus suggest that the increase in plasma uric acid in mice with hepatocyte-specific *G6pc* deficiency enhances atherosclerosis.

Further, the discrepancy between our observations and the limited atherogenesis in GSD Ia patients may be due to the model that we used. Feeding *Ldlr*^*−/−*^ mice WTD is one of the most frequently used models to induce atherogenesis [9]. WTD contains a high percentage of sucrose (35%), and sucrose rapidly increases blood glucose levels, leading to insulin release and hypoglycemia [14]. GSD Ia patients adhere to a strict diet to prevent fasting hypoglycemia [19], including avoiding sucrose intake [20]. The recommended diet for GSD Ia patients consists of 60–70% carbohydrates with a low glycemic index, 10–15% protein, and 15–30% fat [[20], [21], [22]]. We cannot exclude that the continuous low level of blood glucose in our model may have affected cells locally in the vessel wall, and therefore, increased atherogenesis, similar to findings in humans with hypoglycemia [23]. However, elevated plasma TG levels, as observed in GSD Ia patients [3,8,17,24], have, except for one study employing Apolipoprotein C3 (APOC3) overexpression in mice [25], a clear pro-atherogenic role in mice and in humans [[26], [27], [28], [29], [30], [31], [32]]. Since only 9 patients were included in the study on atherogenesis in GSD Ia [5], and in view of the pro-atherogenic role of triglycerides and plasma uric acid, our findings do provide a rationale to investigate atherogenesis in a larger GSD Ia patient cohort.

## Source of funding

M.H. Oosterveer and M. Westerterp are supported by VIDI grants (917.17.373 and 917.15.350, respectively) from the Netherlands Organization of Scientific Research (NWO), and Rosalind Franklin Fellowships from the 10.13039/501100001721University of Groningen.

## CRediT authorship contribution statement

**Anouk M. La Rose:** Conceptualization, Methodology, Investigation, Writing – original draft. **Anouk G. Groenen:** Resources, Writing – review & editing. **Benedek Halmos:** Resources, Writing – review & editing. **Venetia Bazioti:** Resources, Writing – review & editing. **Martijn G.S. Rutten:** Resources, Writing – review & editing. **Kishore A. Krishnamurthy:** Resources, Writing – review & editing. **Mirjam H. Koster:** Resources, Writing – review & editing. **Niels J. Kloosterhuis:** Resources, Writing – review & editing. **Marieke Smit:** Resources, Writing – review & editing. **Rick Havinga:** Resources, Writing – review & editing. **Gilles Mithieux:** Resources, Writing – review & editing. **Fabienne Rajas:** Resources, Writing – review & editing. **Folkert Kuipers:** Conceptualization, Writing – review & editing. **Maaike H. Oosterveer:** Conceptualization, Writing – review & editing. **Marit Westerterp:** Conceptualization, Methodology, Investigation, Writing – review & editing, Supervision, Funding acquisition.

## Declaration of Competing Interest

None.

## References

[1] Lei K.J., Chen Y.T., Chen H., Wong L.J.C., Liu J.L., McConkie-Rosell A. (1995). Genetic basis of glycogen storage disease type 1a: prevalent mutations at the glucose-6-phosphatase locus. Am. J. Hum. Genet..

[2] Chou J.Y., Jun H.S., Mansfield B.C. (2015). Type I glycogen storage diseases: disorders of the glucose-6-phosphatase/glucose-6-phosphate transporter complexes. J. Inherit. Metab. Dis..

[3] Bandsma R.H.J., Prinsen B.H., Van Der Velden M.D.S., Rake J.P., Boer T., Smit G.P.A. (2008). Increased de novo lipogenesis and delayed conversion of large VLDL into intermediate density lipoprotein particles contribute to hyperlipidemia in glycogen storage disease type 1a. Pediatr. Res..

[4] Bandsma R.H.J., Rake J.P., Visser G., Neese R.A., Hellerstein M.K., Van Duyvenvoorde W. (2002). Increased lipogenesis and resistance of lipoproteins to oxidative modification in two patients with glycogen storage disease type 1a. J. Pediatr..

[5] Ubels F., Rake J., Smit P., Smit A., Slaets J. (2002). Is glycogen storage disease 1a associated with atherosclerosis?. Eur. J. Pediatr..

[6] Sacks F.M., Lichtenstein A.H., Wu J.H.Y., Appel L.J., Creager M.A., Kris-Etherton P.M. (2017). Dietary fats and cardiovascular disease: a presidential advisory from the American Heart Association. Circulation.

[7] Lian Z., Perrard X.Y.D., Peng X., Raya J.L., Hernandez A.A., Johnson C.G. (2020). Replacing saturated fat with unsaturated fat in western diet reduces foamy monocytes and atherosclerosis in male Ldlr−/− mice. Arterioscler. Thromb. Vasc. Biol..

[8] Hoogerland J.A., Peeks F., Hijmans B.S., Wolters J.C., Kooijman S., Bos T. (2021). Impaired VLDL catabolism links hypoglycemia to hypertriglyceridemia in GSD Ia. J. Inherit. Metab. Dis..

[9] Daugherty A., Tall A.R., Daemen M.J.A.P., Falk E., Fisher E.A., García-Cardeña G. (2017). Recommendation on design, execution, and reporting of animal atherosclerosis studies: a scientific statement from the American Heart Association. Circ. Res..

[10] Getz G.S., Reardon C.A. (2006). Diet and murine atherosclerosis. Arterioscler. Thromb. Vasc. Biol..

[11] Mutel E., Abdul-Wahed A., Ramamonjisoa N., Stefanutti A., Houberdon I., Cavassila S. (2011). Targeted deletion of liver glucose-6 phosphatase mimics glycogen storage disease type 1a including development of multiple adenomas. J. Hepatol..

[12] Wittenstein B., Klein M., Finckh B., Ullrich K., Kohlschütter A. (2002). Radical trapping in glycogen storage disease 1a. Eur. J. Pediatr..

[13] La Rose A.M., Bazioti V., Hoogerland J.A., Svendsen A.F., Groenen A.G., van Faassen M. (2021). Hepatocyte-specific glucose-6-phosphatase deficiency disturbs platelet aggregation and decreases blood monocytes upon fasting-induced hypoglycemia. Mol. Metab..

[14] Laube H., Schatz H., Nierle C., Fussgänger R., Pfeiffer E.F. (1976). Insulin secretion and biosynthesis in sucrose fed rats. Diabetologia.

[15] Gjorgjieva M., Calderaro J., Monteillet L., Silva M., Raffin M., Brevet M. (2018). Dietary exacerbation of metabolic stress leads to accelerated hepatic carcinogenesis in glycogen storage disease type Ia. J. Hepatol..

[16] Frodermann V., van Duijn J., van Pel M., van Santbrink P.J., Bot I., Kuiper J. (2015). Mesenchymal stem cells reduce murine atherosclerosis development. Sci. Rep..

[17] Bandsma R., Smit P., Kuipers F. (2002). Disturbed lipid metabolism in glycogen storage disease type 1. Eur. J. Pediatr..

[18] Kimura Y., Tsukui D., Kono H. (2021). Uric acid in inflammation and the pathogenesis of atherosclerosis. Int. J. Mol. Sci..

[19] Kishnani P.S., Austin S.L., Abdenur J.E., Arn P., Bali D.S., Boney A. (2014). Diagnosis and management of glycogen storage disease type I: a practice guideline of the American college of medical genetics and genomics. Genet. Med..

[20] Rake J.P., Visser G., Labrune P., Leonard J.V., Ullrich K., Smit G.P.A. (2002). Guidelines for management of glycogen storage disease type I - European study on glycogen storage disease type I (ESGSD I). Eur. J. Pediatr..

[21] Goldberg T., Slonim A.E. (1993). Nutrition therapy for hepatic glycogen storage diseases. J. Am. Diet. Assoc..

[22] Wolfsdorf J.I., Weinstein D.A. (2003). Glycogen storage diseases. Rev. Endocr. Metab. Disord..

[23] Desouza C.V., Bolli G.B., Fonseca V. (2010). Hypoglycemia, diabetes, and cardiovascular events. Diabetes Care.

[24] Rake J.P., Visser G., Labrune P., Leonard J.V., Ullrich K., Smit G.P. (2002). Glycogen storage disease type I : diagnosis, management, clinical course and outcome. Results of the European study on glycogen storage disease type I (ESGSD I). Eur. J. Pediatr..

[25] Ebara T., Ramakrishnan R., Steiner G., Shachter N.S. (1997). Chylomicronemia due to apolipoprotein CIII overexpression in apolipoprotein E-null mice apolipoprotein CIII – induced hypertriglyceridemia is not mediated by effects on apolipoprotein E. J. Clin. Invest..

[26] Masucci-magoulas A.L., Goldberg I.J., Bisgaier C.L., Serajuddin H., Francone O.L., Breslow J.L. (1997). A mouse model with features of familial combined hyperlipidemia. Science (80-).

[27] Li H., Han Y., Qi R., Wang Y., Zhang X., Yu M. (2015). Aggravated restenosis and atherogenesis in ApoCIII transgenic mice but lack of protection in ApoCIII knockouts : the effect of authentic triglyceride-rich lipoproteins with and without ApoCIII. Cardiovasc. Res..

[28] Arca M., Veronesi C., D’erasmo L., Borghi C., Colivicchi F., De Ferrari G.M. (2020). Association of hypertriglyceridemia with all-cause mortality and atherosclerotic cardiovascular events in a low-risk italian population: the tg-real retrospective cohort analysis. J. Am. Heart Assoc..

[29] Jørgensen A.B., Frikke-Schmidt R., West A.S., Grande P., Nordestgaard B.G., Tybjærg-Hansen A. (2013). Genetically elevated non-fasting triglycerides and calculated remnant cholesterol as causal risk factors for myocardial infarction. Eur. Heart J..

[30] Katzmann J.L., Werner C.M., Stojakovic T., März W., Scharnagl H., Laufs U. (2020). Apolipoprotein CIII predicts cardiovascular events in patients with coronary artery disease: a prospective observational study. Lipids Health Dis..

[31] Van Capelleveen J.C., Moens S.J.B., Yang X., Kastelein J.J.P., Wareham N.J., Zwinderman A.H. (2017). Apolipoprotein C-III levels and incident coronary artery disease risk: the EPIC-Norfolk prospective population study. Arterioscler. Thromb. Vasc. Biol..

[32] Sarwar N., Danesh J., Eiriksdottir G., Sigurdsson G., Wareham N., Bingham S. (2007). Triglycerides and the risk of coronary heart disease: 10 158 incident cases among 262 525 participants in 29 Western prospective studies. Circulation.

